# Editorial: *Helicobacter pylori* and its mechanisms of antibiotic survival

**DOI:** 10.3389/fcimb.2023.1164227

**Published:** 2023-02-23

**Authors:** Paweł Krzyżek, Valentina Puca, Rossella Grande

**Affiliations:** ^1^ Department of Microbiology, Faculty of Medicine, Wroclaw Medical University, Wroclaw, Poland; ^2^ Department of Pharmacy, University “G. d’Annunzio” of Cheti-Pescara, Chieti, Italy

**Keywords:** *Helicobacter pylori*, pathogenesis, resistance, tolerance, antibiotics, antimicrobials

*Helicobacter pylori* is one of the most common human pathogens that, in the absence of therapeutic intervention, is able to remain in the gastric environment for an individual’s entire life (Salama et al., 2013). Unfortunately, over time, situations in which treatment does not bring the desired effect are becoming more frequent (Boyanova et al., 2023). In the case of this bacterium, therapeutic failures are very often associated with the presence of genetic mutations at the target sites of antibiotics (Cortés et al., 2021; Roberts et al., 2022). It is increasingly postulated, however, that a variety of physiological processes may also affect the antibiotic tolerance or resistance of *H. pylori*, including biofilm formation (Krzyżek et al., 2020; Hou et al., 2022), morphological transformation (Gladyshev et al., 2020; Krzyżek and Grande, 2020), and secretion of membrane vesicles (Jarzab et al., 2020). With reference to the above, the main purpose of this Research Topic was to draw attention to the importance of resistance and tolerance mechanisms promoting the survival of *H. pylori* under stressogenic conditions, especially antibiotic pressure.

The basis for effectively counteracting antibiotic resistance is a thorough understanding of the factors responsible for the development and spreading of this phenomenon. In a comprehensive review, Liang et al. described strategies for the prevention and effective control of *H. pylori* infections. Attention was drawn to changes in treatment protocols over the last number of decades, including the switch from triple to quadruple therapies, extension of treatment length, and the use of new antibiotics or proton pump inhibitors. The limited effectiveness of these modifications and the need to search for alternative solutions, particularly the inclusion of probiotics in the treatment of *H. pylori* infections, were also highlighted.

Referring to the urgency of applying novel procedures to treat *H. pylori*, in the original article by Wang et al., the effectiveness of liposomal linolenic acid (LipoLLA) against 30 clinical strains of *H. pylori* with different antibiotic resistance profiles was determined. This formulation was found to present a high bactericidal effect (MIC = 3.75–15 µg/mL), with activity dependent on both concentration and incubation time. It was observed that LipoLLA showed synergism or additivity with all four tested antibiotics (amoxicillin, clarithromycin, metronidazole, and levofloxacin), which was most likely related to the ability of LipoLLA to disrupt the continuity of bacterial cell structures and to stimulate increased penetration of these antibiotics. In the last stage of the research, it was shown that LipoLLA had a marginal effect on the tested probiotics and did not disturb the composition of the intestinal microbiota of human volunteers.

A phenomenon of a crucial importance in therapeutic failures, although still poorly verified scientifically, is heteroresistance (a mosaic resistance profile). Therefore, in the next research article of this Research Topic, Wang et al. focused on determining the prevalence and clinical significance of heteroresistance in *H. pylori* strains isolated from gastric biopsies. It was observed that heteroresistance to one of the antibiotics (clarithromycin or levofloxacin) was detected in approximately 14% of strains, and double heteroresistance was identified in more than 3% of isolates. Importantly, it was noticed that most of the strains with levofloxacin heteroresistance presented convergent phenotypic resistance when applying culture identification, whereas the results diverged significantly during analysis of clarithromycin heteroresistance in the same way. The authors pointed out that when using traditional clarithromycin-based treatment methods for *H. pylori*, there is a risk of not detecting heteroresistance to this antibiotic and a possibility of subsequent therapeutic failure.

As stated at the beginning of this Editorial, some complex physiological processes in microorganisms may also generate tolerance or resistance to antimicrobials. Therefore, the next research paper focused on the contribution of biofilm formation in the dissemination of antibiotic resistance in *H. pylori* (Krzyżek et al., 2022). Our research team noticed a strong correlation between clarithromycin resistance or multidrug resistance of *H. pylori* strains and their enhanced biofilm production capacity. It was observed that these strains were characterized by more intensive production of eDNA and proteins, both of which constituted components of the biofilm matrix. The bacterial cells of strong biofilm producers showed an increased tendency for self-aggregation and were more densely entwined with flagella. Additionally, these strains secreted much higher amounts of membrane vesicles into the local environment. These results indicate a close interconnection between the physiological features associated with increased biofilm formation and the antibiotic resistance profile of *H. pylori* strains. It is also worth mentioning that in this article, for the first time, microfluidic conditions were used for comparative analysis of biofilm formation by clinical *H. pylori* strains.

In summary, the articles constituting this Research Topic provide a valuable addition to our understanding of mechanisms responsible for therapeutic failures of *H. pylori* infections. We hope that this knowledge may contribute to the development of new types of therapies targeting *H. pylori* and the reduction of the spread of resistant and multidrug-resistant strains of this bacterium.

## Author contributions

PK wrote the initial draft of the manuscript. All authors have made a substantial, direct, and intellectual contribution to the work and approved it for publication.
